# Virtual Case-Based Learning: Nova Estratégia de Ensino e de Treinamento Médico Digital Humanizado em Cardiologia

**DOI:** 10.36660/abc.20220423

**Published:** 2022-11-09

**Authors:** Manoel Fernandes Canesin, Fabrício Nogueira Furtado, Rodrigo Marques Gonçalves, Diogo Cesar Carraro, Thaísa Mariela Nascimento de Oliveira, Ricardo Rodrigues, Cláudio José Fuganti, Cézar Eumann Mesas, Laércio Uemura

**Affiliations:** 1 Active Metodologias Ativas de Ensino São Paulo SP Brasil Paciente 360, Active Metodologias Ativas de Ensino , São Paulo , SP – Brasil; 2 Departamento de Cardiologia Universidade Estadual de Londrina Londrina PR Brasil Departamento de Cardiologia , Universidade Estadual de Londrina , Londrina , PR – Brasil

**Keywords:** Simulação por Computador, Educação Médica, Aprendizagem, Estudantes de Medicina, Humanização da Assistência

## Abstract

**Fundamento:**

A consolidação de novos paradigmas educacionais exige a implantação de estratégias inovadoras com potencial de transformar estudantes em profissionais competentes.

**Objetivos:**

Analisar o conhecimento e a satisfação de estudantes antes e após a utilização de uma nova metodologia ativa de ensino médico de modelo digital humanizado chamada *Virtual Case-Based Learning* (VCBL).

**Métodos:**

Estudo descritivo com análise documental sobre o processo de ensino-aprendizagem de estudantes de medicina. Dados obtidos da avaliação de conhecimento teórico e do instrumento de satisfação dos alunos nos anos de 2018 e 2019 foram analisados, e a nova metodologia proposta VCBL foi comparada com a metodologia ativa de ensino tradicional, o *Problem-Based Learning* (PBL). As análises descritivas e de associação foram realizadas utilizando o programa Statistical Package for the Social Sciences.

**Resultados:**

Foram analisados 167 documentos aplicados a estudantes de medicina. Em relação à avaliação do conhecimento teórico, os alunos avaliados em 2018 obtiveram média 41,7%, comparados aos alunos de 2019 que alcançaram 73,3% (p<0,001). Entre os estudantes submetidos à avaliação da satisfação com a metodologia de aprendizagem proposta, 76,0% pontuaram o valor máximo para a questão um, e 83,0% para a questão número dois. Cerca de 70,0% dos estudantes classificaram positivamente o aprendizado adquirido após utilização da plataforma Paciente 360; 78,0% responderam que se sentem preparados para o atendimento ambulatorial; e 94,0% pontuaram de forma positiva a metodologia empregada.

**Conclusão:**

Neste estudo inicial, os resultados indicaram que a nova ferramenta em metodologia ativa de ensino médico digital humanizado, o VCBL, pode auxiliar no aprimoramento do processo de ensino-aprendizagem, proporcionando conhecimento e satisfação dos estudantes.

## Introdução

Ao longo dos anos, o *Problem-Based Learning* (PBL), ou aprendizagem baseada em problemas em português, tem sido uma prática pedagógica empregada na educação médica. Este método de ensino-aprendizagem recomenda a realização de atividade guiada por meio de casos clínicos problematizados, e tem por objetivo capacitar os estudantes para discutirem diagnósticos, condutas terapêuticas e outros aspectos do raciocínio clínico enfrentados cotidianamente na profissão. ^1 , 2^

Em consonância com os desafios atuais, sabe-se que a educação médica tem experienciado rápidas mudanças em todo mundo. ^3^ O maior desafio encontrado por docentes está em oportunizar e estimular os estudantes sobre a essência que vai além do raciocínio clínico desenvolvido em sala de aula e laboratórios, ou seja, o vínculo com o paciente. ^4^

Inevitavelmente, nas dependências da universidade, os estudantes são capazes de desenvolver a excelência cognitiva e científica. No entanto, o afeto e a humanização do cuidado só são experimentados quando imersos na prática real. Tradicionalmente, o contexto do atendimento e o contato físico com o paciente têm sido oportunizados somente durante estágios ou internatos. ^1 , 4^

Assim, a consolidação de novos paradigmas educacionais exige a implantação de estratégias que transformem estudantes em profissionais competentes. ^3^ Essa busca permanente tem cooperado para o surgimento de metodologias ativas inovadoras de ensino, aprendizagem e avaliação. ^5^

O método e as fases que compõem a simulação clínica possuem um maior potencial educacional quando comparados aos métodos tradicionais de ensino, no que tange o desenvolvimento do conhecimento e o treinamento de habilidades específicas, devido à oportunidade de vivenciar cenários clínicos simulados, próximos da realidade. ^6 - 8^ No entanto, por se tratar de uma proposta de ensino-aprendizagem presencial, com a utilização de manequins ou pacientes simulados, programas quantitativos e qualitativos de pesquisa são necessários para comprovar os resultados alcançados nos diferentes contextos, para que possam ser replicados e sintetizados na ciência educacional. ^9^

Com base nos paradigmas educacionais e nas necessidades ainda não contempladas, um modelo inédito de aprendizagem simulada muito próximo do real, denominado “ *Virtual Case-Based Learning* (VCBL) *”,* foi desenvolvido e testado. O VCBL oferece uma solução potencial para as limitações de simulações tradicionais, ao considerar o ensino híbrido (presencial e remoto) para uma melhor experiência presencial com o paciente, sem comprometer a sua segurança. Para isso, foi utilizada uma plataforma inovadora de ensino, criada para humanizar a interação digital de aprendizagem. Portanto, o objetivo deste estudo foi avaliar o conhecimento e a satisfação de estudantes de medicina antes e após a utilização de um novo modelo humanizado de metodologia ativa de ensino médico chamado VCBL.

## Métodos

### Delineamento e população do estudo

Trata-se de uma pesquisa exploratória, descritiva e de análise documental. O estudo compreendeu o levantamento e o fichamento de dados referentes ao conhecimento teórico e de um instrumento de autoconfiança e satisfação, aplicados em 167 estudantes do oitavo semestre de medicina em uma universidade pública no sul do Brasil.

**Figura 1:**
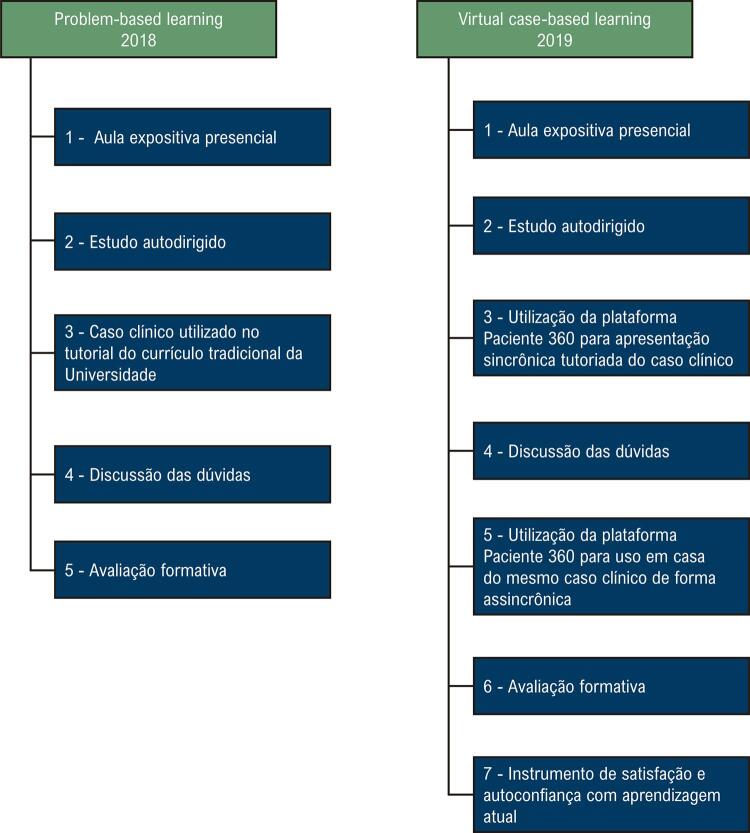
Fluxograma das etapas dos modelos de ensino e documento de análise.

A população de estudo foi dividida em dois períodos, 2018 e 2019. Os alunos que cursaram a disciplina de cardiologia em 2018 foram formados por meio do modelo PBL, utilizado há 20 anos na universidade avaliada (a primeira do Brasil a utilizar o método). Em 2019, o modelo de aprendizagem aplicado foi o VCBL, modelo criado como nova ferramenta de metodologia ativa de ensino. As etapas do protocolo e dos dois modelos de aprendizagem utilizados no estudo estão apresentadas na Figura 1 .

Para os estudantes no oitavo semestre do curso de medicina em 2018, a disciplina de Cardiologia foi oferecida segundo método tradicional de PBL, como a seguir: 1) docente apresenta o caso clínico a ser estudado; 2) os estudantes buscam na literatura o conteúdo necessário e apresentam a resolução do problema. Neste modelo, o docente estimula a tomada de decisão e o raciocínio clínico teórico entre os alunos do grupo, por meio da discussão tutorial e aula expositiva. ^10^ Para a condução da problematização, conforme etapas descritas acima, a turma era dividida em grupos de 10 alunos por estação. As discussões nos pequenos grupos abordavam o material de apoio produzido no programa PowerPoint e o caso clínico em forma de texto.

Em 2019, o grupo de estudantes que passou por essa disciplina foi submetido a essa nova proposta de metodologia ativa de ensino médico, denominada *VCBL* . O método conta com uma plataforma virtual interativa de casos clínicos humanizados, sendo os mesmos casos clínicos discutidos no método PBL (insuficiência coronariana crônica, insuficiência cardíaca crônica, fibrilação atrial, hipertensão arterial e dislipidemia), entretanto, apresentados de maneira simulada interativa humanizada com a utilização da plataforma Paciente 360.

O VCBL compreende as mesmas etapas do PBL, adicionadas as interações com a plataforma Paciente 360 de forma síncrona (com apoio docente) ou assíncrona (sem apoio docente), para a autorreflexão do raciocínio clínico humanizado.

A fim de avaliar o conhecimento cognitivo dos estudantes em ambos os períodos, foi aplicada a mesma avaliação teórica de 25 questões de múltipla escolha. As questões abordaram todo o conteúdo apresentando na disciplina de cardiologia ao longo do módulo, a seguir: insuficiência coronariana crônica e aguda, insuficiência cardíaca crônica e aguda, arritmias, hipertensão arterial e dislipidemia. Portanto, o tema, tempo para finalização, grau de dificuldade e etapa de discussão de dúvidas foram semelhantes entre os períodos estudados. Além disso, os estudantes de 2019, após a avaliação teórica, responderam um instrumento de satisfação sobre o método de ensino VCBL e a utilização da plataforma Paciente 360.

### Ferramenta da metodologia ativa de ensino médico

O VCBL foi aplicado por meio de uma plataforma digital de metodologia ativa de ensino médico, com simulação realística de casos clínicos. A plataforma apresenta casos clínicos com pessoas reais, e permite ao estudante a interação e tomada de decisão em todas as etapas de uma consulta médica em diferentes temas e especialidades. Assim, a ferramenta proporciona, de forma humanizada, interativa e inovadora, a empatia e a afetividade para a aprendizagem de ensino médico.

A plataforma chamada Paciente 360 foi desenvolvida com o objetivo de auxiliar na melhoria da qualidade acadêmica do ensino médico e permitir melhor conexão acadêmica com as novas gerações de alunos. É utilizada desde 2019 em universidades dentro e fora do Brasil.

**Figura 2:**
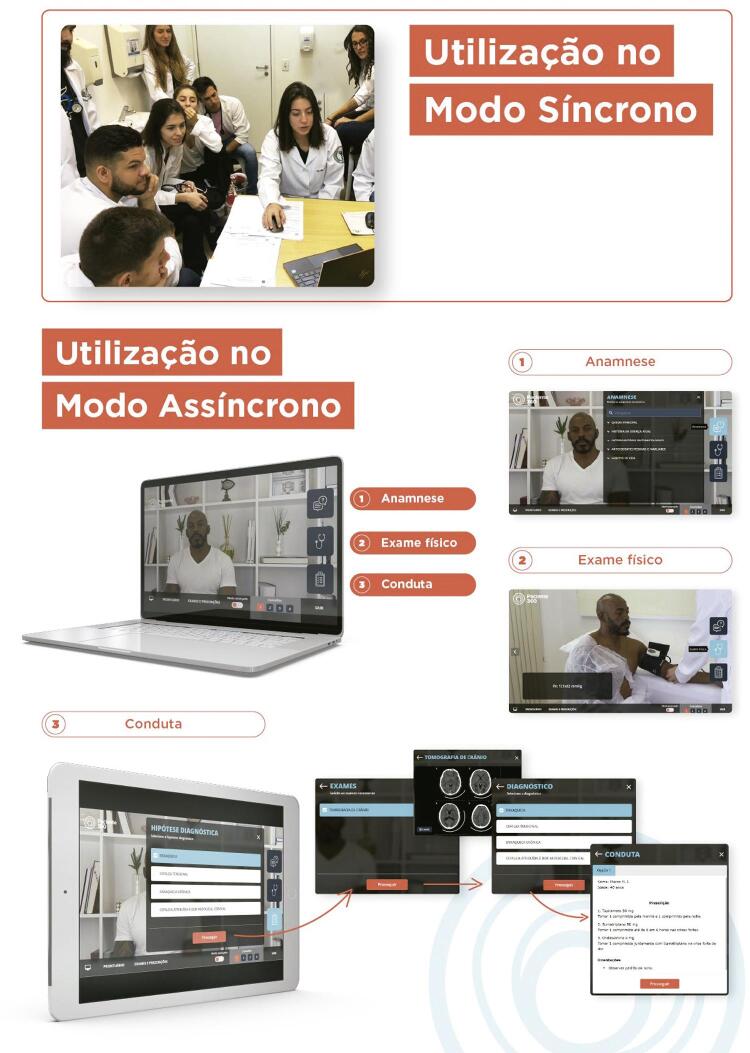
Uso síncrono e assíncrono da ferramenta utilizada no Virtual Case-Based Learning (VCBL).

No módulo assincrônico, o aluno, de casa ou de qualquer outro local, sem ajuda de um professor ou tutor, pode atender pacientes com diferentes doenças simuladas, realizar a anamnese, o exame físico completo, solicitar e analisar os resultados de exames laboratoriais e de imagem, dar o diagnóstico e, ao final, escolher a conduta que melhor se aplica para o caso ( Figura 2 ). O docente tutor dá *feedback* de acertos e erros, e pode ainda, pelo módulo sincrônico, apresentar o caso clínico e realizar a discussão de todas as etapas com grupos de alunos.

### Coleta de dados

A avaliação teórica foi composta por 25 questões de múltipla escolha e avaliou o conhecimento cognitivo dos estudantes no ano de 2018 e 2019.

O instrumento de satisfação e autoconfiança com a aprendizagem atual, aplicado em 2019, foi composto por cinco questões *likert* , construídas pelos docentes da disciplina de cardiologia da mesma universidade.

A satisfação com a aprendizagem atual foi avaliada por meio de duas perguntas com pontuação de 0 a 10: 1) “Em uma escala de 0 a 10, qual a chance de você indicar o Paciente 360 para um amigo?”; e 2) “Em uma escala de 0 a 10, como você classifica a metodologia de casos clínicos interativos humanizados VCBL utilizada no atual módulo de Cardiologia em relação à metodologia tradicional de casos clínicos do PBL utilizada nos módulos anteriores do mesmo período (nefrologia e pneumologia)?

Além disso, três questões classificavam como “pouco, satisfatório, bom, muito bom e excelente” o ganho da autoconfiança: 3) “Como você avalia seu aprendizado após o uso do Paciente 360?”; 4) “Você se sente mais preparado para o atendimento ambulatorial?”; e 5) “Como você avalia o conteúdo discutido?”.

Para a coleta de dados, foi construído um instrumento para a identificação, organização e fichamento da pontuação individual da avaliação teórica aplicada em 2018 e 2019, e do instrumento de satisfação aplicado somente em 2019. Utilizou-se as etapas propostas pela literatura, ^11^ como a apuração e organização do material disponível, interpretação dos dados e análise crítica dos documentos.

### Análise estatística

A análise descritiva foi realizada por meio de frequências absolutas e relativas das variáveis categóricas e, para as variáveis contínuas foram calculadas médias e desvios-padrão.

As comparações entre médias de variáveis contínuas foram analisadas pelo teste t de Student após confirmação da distribuição normal pelo teste de Kolmogorov-Smirnov.

Os dados foram analisados usando o software *Statistical Package for the Social Sciences* (IBM SPSS Statistics for Windows, Versão 20.0. Armonk, NY: IBM Corp.). Para todas as análises, foi considerado um nível de significância estatística de p<0,05.

### Aspectos éticos

O comitê de ética em pesquisa envolvendo seres humanos da Universidade Estadual de Londrina foi consultado para a produção do presente estudo, e este foi liberado sem necessidade de uso do consentimento informado, pois todos os participantes foram informados sobre o objetivo da pesquisa e receberam garantia de anonimato.

## Resultados

Foram analisadas 87 avaliações teóricas formativas obrigatórias, referentes à turma de cardiologia de 2018. Em 2019, 80 avaliações teóricas foram analisadas e, destes, 17,5% perderam o prazo de sete dias para preenchimento do instrumento sobre satisfação com o modelo VCBL como metodologia ativa de ensino ( Quadro 1 ). A comparação incluindo os alunos não respondentes estão representados no material suplementar ( Tabela S1 ).

**Quadro 1:** Descrição da população de estudo segundo dados documentais e modelo de aprendizagem

Turma	Número de estudantes	Modelo de aprendizagem	Documento de análise
2018	87	Problem-Based Learning	Avaliação teórica
2019	80	Virtual Case-Based Learning	Avaliação teórica Instrumento de satisfação e autoconfiança com a aprendizagem atual

**Figura 3:**
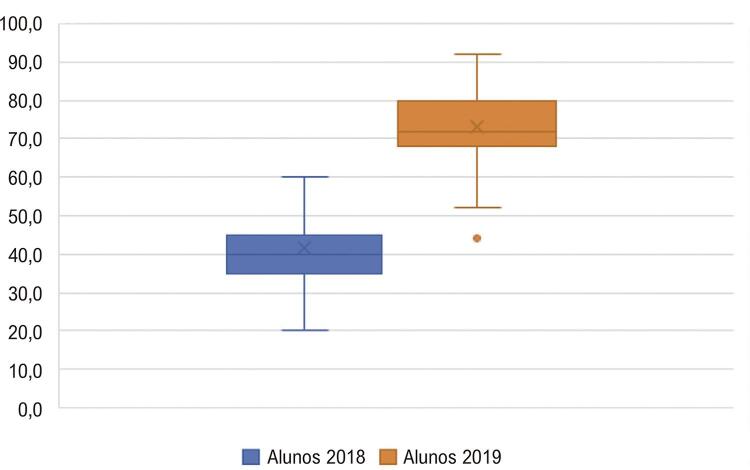
Comparação do percentual médio de acertos da avaliação de conhecimento teórico de alunos de medicina, 2018 e 2019.

A Figura 3 apresenta a comparação da média percentual da avaliação do conhecimento teórico. Os alunos de 2018 obtiveram uma média 41,7%, variando de 20,0% a 60,0%, e os alunos de 2019 alcançaram a média 73,3%, com variação de 44,0% a 92,0% (p <0,001).

**Figura 4:**
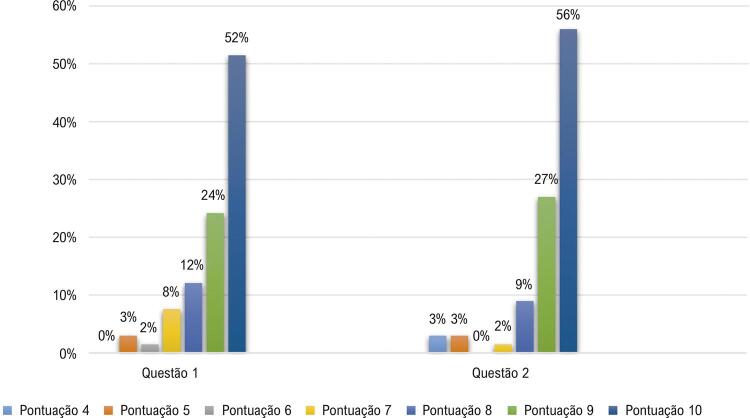
Satisfação com a aprendizagem atual de alunos de medicina, 2019.

Quanto a satisfação com a aprendizagem atual, 76,0% dos estudantes avaliaram com pontuação máxima (9-10) a questão um e 83,0% a questão dois, conforme Figura 4 .

**Figura 5:**
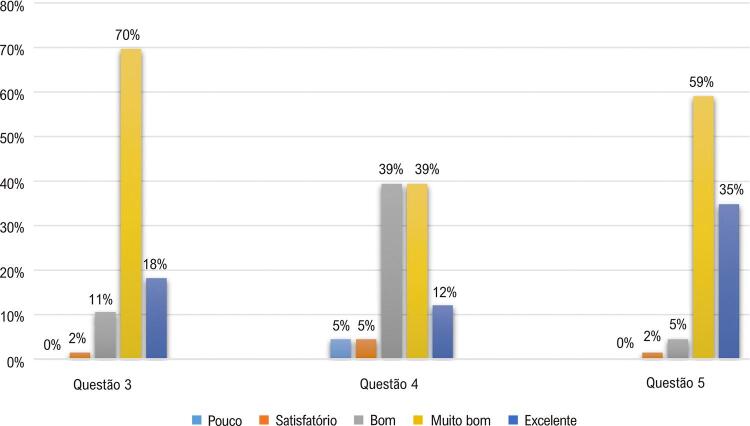
Autoconfiança na aprendizagem e avaliação da nova proposta de ensino de alunos de medicina, 2019.

Cerca de 70,0% dos estudantes classificaram como “muito bom” o aprendizado adquirido após utilização da plataforma Paciente 360; 78,0% julgaram como “bom” e “muito bom” o sentimento de estarem preparados para o atendimento ambulatorial; e 94,0% avaliaram como “muito bom” e “excelente” a abordagem do conteúdo, por meio da nova proposta de aprendizagem ( Figura 5 ).

## Discussão

Ao ingressar no campo clínico, os estudantes de medicina deparam-se com inúmeras condições que exigem a aplicação integrada do conhecimento teórico e habilidades práticas, associada ao desenvolvimento da humanização e empatia com o paciente para a garantia de um cuidado integral. ^12^ Estudos corroboram ^12 , 13^ que modelos tradicionais de ensino-aprendizagem não têm atendido aos requisitos do ambiente contemporâneo da realidade médica, em que há uma lacuna entre a formação e a prática clínica integral humanizada.

Atualmente, a simulação realística tem sido utilizada por várias universidades, com o intuito de formar profissionais que contemplem as exigências do mercado de trabalho. ^6 , 8 , 14^ A maior parte delas com manequins de simulação não humanizados ou avatares. Autores de um estudo ^15^ recente revelaram uma limitação deste método, ao concluir que as fases da simulação realística não permitem ao estudante o desenvolvimento da empatia e a socialização com paciente real, e propuseram que novos métodos sejam criados com esse objetivo.

O presente estudo apontou a viabilidade e eficácia do novo modelo proposto de aprendizagem simulada para que outras universidades de medicina possam replicá-lo. Este método mostrou-se eficaz na avaliação formativa de conhecimento teórico da disciplina de cardiologia. A pontuação média obtida pelos alunos de 2019 foi superior à pontuação dos alunos de 2018 em mais de 30,0%, mostrando que o processo de ensino-aprendizagem foi potencializado após a experiência com as etapas propostas pelo método VCBL.

Tecnologias de simulação integrada está passando por um rápido desenvolvimento. O ensino médico digital está desempenhando um papel cada vez mais importante no treinamento do conhecimento e habilidades clínicas para estudantes de medicina. ^13^ Atualmente, nenhuma simulação retrata de forma realista todos os componentes fisiológicos, mentais e comportamentais do atendimento ao paciente. ^16^ Por isso, o reconhecimento da autoconfiança e da satisfação dos alunos ao participar de novas estratégias contribui para o aperfeiçoamento destas.

Todos os estudantes desta pesquisa indicariam a plataforma Paciente 360 para um amigo. Destes, 76,0% optaram pelas opções de maiores pontuações (9-10) do instrumento de satisfação. Aproximadamente 90,0% dos indivíduos classificaram como “muito bom” e excelente” o aprendizado adquirido após utilização da plataforma, resultado no aprimoramento da autoconfiança dos estudantes.

As etapas três e cinco ( Figura 1 ) da metodologia VCBL são consideradas o “coração” da nova proposta metodológica. Ela utiliza a nova plataforma como ferramenta em metodologia ativa de ensino, focando em uma discussão de casos clínicos humanizados interativos inicialmente tutoreada pelo docente (síncrona) e posteriormente realizada como reforço pelo aluno em formato de classe de aula invertida (assíncrona), garantindo um aprendizado realístico mais profundo e em multietapas.

Este *software* de aprendizagem interativa, possibilita o contato virtual, presencial ou remoto com um paciente simulado durante a anamnese, exame físico, exames complementares e conduta. A realização virtual do exame físico possibilita a simulação da inspeção, palpação, percussão e ausculta de todos os sistemas do corpo humano. Além disto, durante a consulta médica simulada, o estudante será capaz de propor hipóteses diagnósticas, solicitar e obter resultados de exames, e planejar a conduta adequada para resolução do caso. O docente, da mesma forma, pode utilizar a ferramenta de modo sincrônico para as etapas de discussão tutorial em grupo.

A autoconfiança é considerada um indicador de proatividade nas situações clínicas para o desfecho de sucesso. Por isso, o profissional deve se sentir capaz de atuar de forma adequada, caso contrário, podem ocorrer atrasos desnecessários no atendimento, aumento no nível de ansiedade e no número de erros. ^10 , 17^

Mais de 80,0% dos estudantes classificaram com maiores pontuações (9-10) a metodologia de ensino utilizada no módulo estudado de cardiologia em comparação à metodologia utilizada nos módulos anteriores. Aproximadamente 80,0% dos estudantes avaliaram como “bom” ou “muito bom” o sentimento de estarem preparados para o atendimento ambulatorial, e 94,0% pontuaram que a abordagem do conteúdo neste formato foi muito boa ou excelente.

Os resultados desta pesquisa corroboram trabalhos científicos que utilizaram a proposta VCBL. O uso da estratégia proporciona a imersão e aproximação do público ao tema, e amplia o acesso à educação em saúde por meio de interações reais e humanizadas. ^18 , 19^ Além disso, após atividade piloto de prática de atendimento clínico avaliando um paciente virtual referindo uma queixa cardiológica, os alunos apresentaram 70,0% de reações positivas no *Net Promoter Score* . Ambos os estudos afirmam que a plataforma Paciente 360 é um modelo de ensino adequado para a realização da educação médica continuada e humanizada em cardiologia, pois promoveu alto grau de satisfação dos participantes, percepção de aquisição de conhecimento e preferência pelo modelo digital de discussão de casos clínicos.

Algumas limitações metodológicas devem ser abordadas para a correta interpretação dos resultados deste estudo. Os dados do ano de 2018 foram coletados retrospectivamente e, no período, o único instrumento de avaliação disponível era a avaliação teórica. Em 2019, o mesmo método avaliativo foi utilizado, entretanto, com adição de instrumentos de satisfação e autoconfiança. Portanto, foi possível realizar análises comparativas importantes e adicionar dados diferenciais ao método inédito VCBL. Ainda, embora não haja uma medida direta de quanto o método tenha contribuído para o conhecimento, uma vez que as notas mais elevadas podem ser decorrentes de outros processos institucionais, o uso da plataforma proporcionou aos alunos um alto grau de satisfação e a oportunidade de uma inserção simulada, realística e humanizada em casos clínicos, possivelmente responsável pelo aumento do engajamento e interesse dos alunos na disciplina de cardiologia. Ainda assim, é importante destacar que o termo humanização é tratado de maneira polissêmica na literatura científica, e esta nova proposta estratégica pedagógica pode ser utilizada com o propósito de promover a humanização na educação médica brasileira.

## Conclusão

O presente estudo indicou melhora no processo de ensino-aprendizagem de estudantes de medicina após a utilização do modelo VCBL em comparação ao método tradicional PBL, mesmo com as limitações apresentadas no estudo. Além disto, foi demonstrada uma grande satisfação dos estudantes ao utilizarem a nova ferramenta em metodologia ativa de ensino médico chamada plataforma Paciente 360. O *software* proporcionou uma aprendizagem humanizada, imersiva e realista.

Embora sejam necessárias mais pesquisas para creditar a eficácia da estratégia de ensino e da ferramenta utilizada, espera-se que este modelo, embasado na metodologia ativa de ensino médico voltada para a geração X, Y e Z, possa fomentar em diferentes universidades a implantação do método e a criação de outros similares. Portanto, a fim de auxiliar na formação de currículos médicos melhores e mais atualizados, os estudantes devem ter oportunidades ampliadas para experienciar o ensino simulado, interativo, digital e humanizado.

## * Material suplementar

Para informação adicional, por favor,clique aqui.
