# Supplementary material for: Virtual Case-Based Learning: Nova Estratégia de Ensino e de Treinamento Médico Digital Humanizado em Cardiologia
Source: Arq Bras Cardiol. 2022 Nov 9;119(5 Suppl 1):35–42. [Article in Portuguese] doi: 10.36660/abc.20220423 (PMC9750197; doi:10.36660/abc.20220423)
Supplement: Translation

# Translation: Learning: New Strategy for Humanized Digital Medical Education and Training in Cardiology

**Authors:** Manoel Fernandes Canesin, Fabrício Nogueira Furtado, Rodrigo Marques Gonçalves, Diogo Cesar Carraro, Thaísa Mariela Nascimento de Oliveira, Ricardo Rodrigues, Cláudio José Fuganti, Cézar Eumann Mesas, Laércio Uemura

**Affiliations:** 1 Brazil Paciente 360, Active Metodologias Ativas de Ensino, São Paulo, SP – Brazil; 2 Brazil Departamento de Cardiologia, Universidade Estadual de Londrina, Londrina, PR – Brazil

**Keywords:** Computer Simulation, Education, Medical, Aprendizagem, Students, Medical, Humanization of Assistance

## Abstract

**Background:**

The consolidation of new educational paradigms requires the implementation of innovative strategies to transform students into competent professionals.

**Objectives:**

To assess knowledge and satisfaction of medical students before and after the use of a new humanized digital model of active learning, called virtual case-based learning (VCBL).

**Methods:**

This was a descriptive, documentary analysis of the teaching-learning process of medical students. Data obtained from theoretical knowledge assessment and satisfaction evaluation questionnaires applied in 2018 and 2019 were analyzed, and the new VCBL was compared with the traditional active methodology PBL (problem-based learning). Descriptive and association analyses were made using the Statistical Package for the Social Sciences.

**Results:**

A total of 167 evaluation forms administered to medical students were analyzed. In the evaluation of theoretical knowledge, the 2018 and the 2019 student groups had a mean of 41.7% and 73.3%, respectively (p<0.001). Among the students who responded to the satisfaction evaluation form, 76.0% gave the highest rating to question one, and 83.0% to question two. Nearly 70.0% of students positively evaluated knowledge acquisition with the Paciente 360 platform; 78.0% reported to feel prepared for working in outpatient care; and 94.0% positively evaluated the new method.

**Conclusion:**

In this initial study, the results indicate that the new active method for humanized digital medical education, the VCBL, can help in the betterment of the teaching-learning process, promoting knowledge and satisfaction by the students.

## Introduction

Problem-based learning (PBL) is a pedagogical approach that has been used in medical education over the years. This teaching-learning method recommends activities guided by clinical cases as trigger problems, and is aimed at capacitating students to discuss diagnoses, therapeutic decisions, and other aspects of clinical reasoning faced by physicians in daily practice.^1 , 2^

In consonance with current challenges, medical education has experienced rapid changes in all the world.^3^ The biggest challenge for physicians has been to create opportunities and stimulate student’s interest for an essence that goes beyond clinical reasoning discussed in the classrooms or labs, that is, the bond with patients.^4^

In the university facilities, students can inevitably develop cognitive and scientific excellence. However, affection and humanization of care can only be experienced during actual practice. For example, opportunities of face-to-face consultations and physical contact with patients are only offered during internships.^1 , 4^

Therefore, consolidation of new educational paradigms requires implementation of strategies that transform students into competent professionals.^3^ This continuous search has contributed to the emergence of innovating, active methodologies of teaching, learning and assessment.^5^

The method and the stages of clinical simulation have a greater educational potential as compared with conventional teaching methods, regarding the development of knowledge and training of specific abilities, due to the opportunities of experiencing reality-like, simulated clinical scenarios.^6 - 8^ However, as it consists of an in-person teaching-learning proposal, including the use of manikins or simulated patients, clinical simulation requires quantitative and qualitative programs to confirm the results obtained in different contexts before replicating and synthesizing them in educational science.^9^

Based on educational paradigms and unmet needs, an original model of simulation-based learning, named “virtual case-based learning (VCBL) *”,* was launched. VCBL offers a potential solution for limitations of conventional simulation-based methods, as it considers blended learning (in-person and online learning) for a better in-person experience with the patient, without compromising patient safety. For this purpose, an innovative learning platform was used, to humanize digital interaction of learning. Thus, the objective of this study was to assess knowledge and satisfaction of medical students before and after the implementation of the new humanized model of active learning method called VCBL.

## Methods

### Study design and population

This investigation was an exploratory, descriptive, and documentary analysis. The study consisted of collecting and registering of data obtained from a theoretical knowledge assessment, and from an instrument for self-confidence and satisfaction evaluation, administered to 167 students in the eighth semester of medical school in a public university in the south of Brazil.

The study population was divided into two periods, 2018 and 2019. In 2018, the students took the course of Cardiology through the PBL method, which has been used in the university for 20 years (the first university to use PBL in Brazil). In 2019, the VCBL was applied, which was proposed as a new active learning method. The steps of the study protocol and of the two learning models analyzed in the study are presented in Figure 1 .

**Figure 1:** Flowchart of the learning models’ steps and the document of analysis..

For the students in the eighth semester of the medical course in 2018, the course of Cardiology was offered following the traditional PBL method, as follows: 1) the teacher presents the clinical case; 2) the students search the content in the literature and present the problem solution. In this model, the teacher stimulates the decision-making process among the students by tutorial discussion and expository class.^10^ The students were divided into groups of 10 to work and solve the problem; discussions were made based on support materials created using PowerPoint, and the clinical case described in a text.

In 2019, the group of students that took the discipline of Cardiology underwent this new proposal of active learning method, called VCBL. This VCBL model has an interactive virtual platform of humanized clinical cases, which were the same as those discussed in the PBL method (chronic coronary artery disease, atrial fibrillation, arterial hypertension, and dyslipidemia), but presented in a humanized interactive simulation, through the *Paciente 360* platform.

The VCBL method has the same stages as the PBL method, in addition to synchronous interactions with the *Paciente 360* platform (with the teacher support) or asynchronous (without the teacher support) for self-reflection of humanized clinical thinking.

To assess students’ cognitive knowledge, a theoretical test with 25 multiple choice questions was administered to both groups (2018 and 2019). The questions addressed the topics covered in Cardiology over the semester, as follows: acute and chronic coronary disease, arrythmias, arterial hypertension and dyslipidemia. Thus, the subject, the time to conclude the test, the difficulty level, and the stage for clarification of doubts were similar between the study periods. In addition, the students of 2019 completed an instrument of satisfaction about the VCBL method and the use of the *Paciente 360* platform.

### Instrument for the active approach to medical education

The VCBL was applied through an online platform of active medical education, with realistic simulation of clinical cases. The platform presents clinical cases with real people and enables the student to interact and make decisions in all stages of a medical consultation in different subjects and specialties. Therefore, the instrument promotes empathy and affection for medical learning, in a humanized, interactive and innovative way.

The *Paciente 360* platform was developed aiming at improving academic quality of medical education and academic connection with the incoming generations of students. The platform has been used in universities in Brazil and other countries since 2019.

In the asynchronous method, the students, from their homes or any other place, without the help from teachers or tutors, can assist patients with different simulated diseases, take medical history, perform physical examination, order and analyze laboratory and imaging tests, make the diagnosis and choose the best treatment for the case ( Figure 2 ). The teacher gives feedback on correct and incorrect decisions to the students; also, through the synchronous mode, the teacher can present the clinical case and promotes discussions about all steps among the students of the group.

**Figure 2:** Use of the synchronous and asynchronous modes of the virtual case-based learning (VCBL) method.

### Data collection

The theoretical exam was composed of 25 multiple-choice questions and evaluated cognitive knowledge of students in 2018 and 2019.

The instrument that evaluated students’ satisfaction and self-confidence with current learning, applied in 2019, was composed of five Likert-scale questions, constructed by teachers of Cardiology of the same university.

Satisfaction with current learning was assessed by two 10-point Likert scale: 1) In a 0-10 point scale, what is the likelihood of recommending *Paciente 360* to a friend?”; and 2) In a 0-10 point scale, how do you classify the VCBL method for humanized, interactive clinical cases, currently used in the course of Cardiology, as compared with the traditional PBL method used in previous courses (Nephrology and Pneumology) during the same period?

In addition, three questions evaluated the level of self-confidence (a little confident, slightly confident, somewhat confident, quite confident, extremely confident): 3) “How do you evaluate your learning progress with *Paciente 360* ?”; 4) “Do you feel more prepared for working in outpatient care?”; and 5) “How do you evaluate the topics discussed?”.

For data collection, an instrument was constructed to identify, organize and register individual ratings obtained from the students in the theoretical test administered in 2018 and 2019, and in the questionnaire on satisfaction administered in 2019. The steps proposed in the literature were used,^11^ including the analysis and organization of the materials available, data interpretation and critical analysis of the documents.

### Statistical analysis

Descriptive analysis of data was made by absolute and relative frequencies of categorical variables, and mean and standard deviation of continuous variables.

Comparisons between mean and continuous variables were analyzed by the Student’s t-test after the normal distribution of data was confirmed by the Kolmogorov-Smirnov test.

Analysis was made using the Statistical Package for the Social Sciences (IBM SPSS Statistics for Windows, Version 20.0. Armonk, NY: IBM Corp.). A p<0.05 was considered statistically significant.

### Ethical aspects

The human research ethics committee of the State University of Londrina was consulted for the development of the present study. The informed consent was waived, as all participants were informed about the objective of the study and had the guarantee of anonymity.

## Results

A total of 87 theoretical, formative evaluations applied in 2018 were examined. For the 2019 group, 80 theoretical tests were analyzed, and 17.5% of the students missed the seven-day deadline to fill up the questionnaire about satisfaction with the use of the VCBL model as an active learning method ( Chart 1 ). The comparison including the non-respondents is presented in supplementary material ( Table S1 ).

**Chart 1:** Study population by instrument of evaluation and learning method

Year	Number of students	Learning model	Evaluation instrument
2018	87	Problem Based Learning	Theoretical evaluation
2019	80	Virtual Case Based Learning	Theoretical evaluation Evaluation instrument for satisfaction, self-confidence and learning progression

Figure 3 shows the comparison of theoretical knowledge evaluated. The 2018 students had a mean of 41.7% (20-60% variation) of correct answers, and the 2019 students had a mean of 73.3% (44.0-92.0% variation) (p<0.001).

**Figure 3:** Comparison of mean percentage of correct answers in the evaluation of theoretical knowledge between the 2018 and the 2019 medical student groups.

With respect to satisfaction with current learning, 76.0% of the students gave the highest rating (9-10) to question number one, and 83.0% to question number two ( Figure 4 ).

**Figure 4:** Satisfaction ratings to learning progression by medical students, 2019.

Nearly 70.0% of the students felt “quite confident” about the learning acquired after using the *Paciente 360* platform; 78.0% felt “somewhat confident” or “quite confident” in working in the outpatient care; and 94.0% felt “quite confident” and “extremely confident” about the content approach through the new learning proposal ( Figure 5 ).

**Figure 5:** Self-confidence about learning progression through the use of the new learning method proposed to medical students in 2019.

## Discussion

When entering the clinical field, medical students face several situations that require the integrated application of theoretical knowledge and practical abilities, associated with the development of humanization and empathy with patients to guarantee integral care.^12^ Studies^12 , 13^ have corroborated that traditional teaching-learning methods have not fulfilled the requirements of contemporary medical environment, with a gap between formal education and humanized, integrated practice.

Today, realistic simulation has been used in many universities, in attempt to train professionals that meet the demand of current labor market.^6 , 8 , 14^ Most methods are based on non-human simulation with manikins or avatars. A recent study^15^ reported a limitation of these methods, as the stages of realistic simulation do not enable students to develop empathy and to socialize with the real patients. The authors suggest the need for new methods to address these objectives.

The present study demonstrated the feasibility and efficacy of the new simulation model of learning proposed so that other universities can use it. This method was shown to be effective in the formative evaluation of theoretical learning in the discipline of Cardiology. Mean ratings obtained from the 2019 student group were more than 30.0% higher than those from the 2018 group, indicating that the teaching-learning process was potentiated by the steps proposed by the VCBL method.

Integrated simulation technologies have been through rapid development. Digital medical education has played a more and more important role in the training of medical students in clinical knowledge and abilities.^13^ Today, there is no simulation method that depicts, in a realistic manner, all physiological, mental and behavioral components of patient care.^16^ Thus, recognizing satisfaction and self-confidence of students in participating of new education strategies contribute to their improvement.

All students who participated in this study would recommend the *Paciente 360* platform to a friend. Of all participants, 76.0% rated the highest scores (9-10) in the Likert scale for satisfaction. Approximately 90.0% of the individuals answered to feel “quite confident” or “extremely confident” about learning progression with the platform, resulting in higher level of self-confidence among students.

Steps three and five ( Figure 1 ) of the VCBL method are considered the “core” of the new methodology. In VCBL, the platform is used as a tool in the active methodology, focusing on discussion of humanized, interactive, clinical cases, first guided by teachers (synchronous mode), then held and reinforced by the students in an inverted class fashion (asynchronous), ensuring a more profound and multistage realistic learning.

This interactive learning software allows virtual, face-to-face or remote contact with a simulated patient during medical history taking, physical examination, complementary tests and decision-making. Virtual physical examination allows simulation of inspection, palpation, percussion, and auscultation of all body systems. Besides, during simulated consultation, the student can suggest diagnostic hypotheses, order and obtain results of complementary tests and plan the most adequate treatment to the case. Likewise, the teacher can use this tool in the steps of tutorial discussion with their students in a synchronous mode.

Self-confidence is considered an indicator of proactivity in clinical situations for a successful outcome. For this reason, professionals must feel confident to act in an assertive way, and avoid unnecessary delays in health care, and increased anxiety and errors.^10 , 17^

More than 80.0% of the students rated the highest scores (9-10) the learning method used in the course of Cardiology as compared with the method used in previous courses. Nearly 80.0% of students answered to feel “somewhat confident” or “quite confident” in working in outpatient care, and 94.0% classified the content approach strategy in the VCBL as “very good” or “excellent.

Results of the present study corroborate previous scientific studies in which VCBL was used. This strategy promotes the immersion and proximity of the public into the themes and expands the access to health education through real and humanized interactions.^18 , 19^ In addition, 70.0% of students gave positive feedback in the Net Promoter Score to a pilot practical activity of clinical care in which a virtual patient, referred for cardiological symptoms. Both studies confirm that the *Paciente 360* platform is an appropriate teaching model for continuing and humanized medical education in cardiology, as the instrument promoted high degree of satisfaction, perception of knowledge acquisition, and preference for the digital model of clinical case discussion.

Some methodological limitations should be addressed for correct interpretation of our results. Data from the year 2018 were collected retrospectively, and in this period, the only evaluation tool available was the theoretical assessment. In 2019, the same evaluation method was used, but with the addition of instruments on satisfaction and self-confidence. Therefore, we were able to perform comparative analyses and add differential items to this original, VCBL method. Also, although there is not a direct measure of the contribution of this method to knowledge, since the highest scores may be resultant from other institutional processes, there was a high degree of satisfaction with the platform, and the opportunity of a simulated, realistic and humanized immersion into the cases, possibly responsible for increased engagement and interest by the students. It is also important to mention that the word “humanization” has been considered as a polysemic term in scientific literature, and this new pedagogical strategy may be used with the aim of promoting humanization in Brazilian medical education.

## Conclusion

The present study indicated an improvement in the teaching-learning process of medical students after the use of the VCBL model as compared with the traditional PBL method, even with limitations of the study. There was a high degree of satisfaction towards this new platform of active methodology of medical education called *Paciente 360* . This software promoted a humanized, immersive and realistic learning.

Although further research is needed to confirm the efficacy of this learning strategy and tool, we expect that this model, based on active methodology of medical education to the X, Y and Z generations, may foster the implementation of the method and creation of similar ones in different universities. Thus, to help in the construction of better and updated medical curricula, the students should have expanded opportunities to experience a simulated, interactive, digital and humanized type of education.

## * Supplemental Materials

For additional information, please click here.
